# Empiric antibiotic therapy resistance and mortality in emergency department patients with bloodstream infection: a retrospective cohort study

**DOI:** 10.1186/s12873-025-01177-0

**Published:** 2025-01-27

**Authors:** Leonhard M. von Beck, Gabriella Anna Rapszky, Veronika E. Kiss, Szilard Sandor, Szabolcs Gaal-Marschal, Tamas Berenyi, Csaba Varga, Bank G. Fenyves

**Affiliations:** Department of Emergency Medicine, Semmelweis University, Ulloi ut 78/A, Budapest, 1082 Hungary

**Keywords:** Sepsis, E. Coli, S. Aureus, East-Central Europe, Hungary

## Abstract

**Background:**

Timely management of sepsis in the emergency department, including the use of appropriate antimicrobials, is crucial for improving patient outcomes. Inadequate empiric antimicrobial treatment is associated with potential changes in patient outcomes. We aimed to pinpoint risk factors, characterize antibiotic resistance trends, and investigate the association between antibiotic resistance and mortality among patients with bacteremia admitted to the emergency department.

**Methods:**

We conducted a retrospective analysis of emergency department patients admitted between 15/06/2016 and 30/09/2022. Patients with a positive blood culture receiving emergency department-initiated antibiotic therapy were included. Antibiotic administration, resistance, and survival data were collected. Descriptive statistics, survival analysis, and Cox proportional hazards models were performed.

**Results:**

Of 157,884 emergency department visits, 1,136 patients had a positive blood culture and received antibiotic therapy initiated in the emergency department. Resistance against empiric antibiotics was 14.5%. The overall 30-day and one-year mortality was 38.6% and 61.8%, respectively. In adjusted Cox models, patients with *Escherichia coli* or *Staphylococcus aureus* infection had 36% lower and 44% higher risk of death, respectively. Although resistance to emergency department-administered antibiotic therapy was not associated with overall mortality, one-year mortality of patients with *Escherichia coli* bacteremia was higher in those with antibiotic resistance (69.0% vs. 49.4%, *p* = 0.011); these patients had a 1.5-fold increased risk of death in an adjusted Cox model.

**Conclusion:**

The mortality of patients with bloodstream infection is high. The association of mortality with empiric emergency department-initiated antibiotic adequacy is pathogen-dependent.

**Supplementary Information:**

The online version contains supplementary material available at 10.1186/s12873-025-01177-0.

## Background

Sepsis is a complex, infection-induced organ dysfunction syndrome with a generally poor outcome. If not recognized early, it can rapidly progress to septic shock, multiorgan failure, and eventually death [1]. Sepsis is associated with the death of 5.3 million people worldwide every year [1, 2]. Each hour of delay in adequate antimicrobial therapy decreases survival in patients with septic shock [3], therefore, timely recognition, diagnosis, and treatment are crucial.

Early management with adequate antimicrobials and rapid resuscitation strategies has been shown to be associated with improved patient outcomes [4]. Thus, emergency departments (EDs) play a critical role in sepsis management [5, 6]. Although early empiric antimicrobial therapy is a cornerstone of treatment, the widespread use of broad-spectrum antibiotics may increase antibiotic resistance [7], which significantly impacts patient outcomes [8, 9]. While the importance of early empiric therapy is well recognized, there is conflicting evidence regarding whether resistance to ED-initiated empiric antibiotics is associated with patient outcomes [8, 10–13]. Moreover, despite its importance, only limited analyses are available on antibiotic resistance-dependent outcomes of septic patients presenting to the ED in the East-Central European region.

To address this gap, and considering the controversial evidence in the literature, we aimed to further investigate the association between resistance to ED-administered empiric antibiotic therapy and mortality.

## Methods

### Study population

This single-center retrospective study was conducted between 15/06/2016 and 30/09/2022 at the Emergency Department of Semmelweis University, Budapest, Hungary. The university hospital has 3000 beds in 40 departments, covers the entire spectrum of medical care and provides healthcare services for over 2,500,000 cases annually. The ED treats non-traumatic emergencies, with an increasing annual patient volume (11,000 visits in 2017, 38,000 in 2022). In this study, patients who underwent blood culture sampling in the ED were included. From these, cases with positive blood culture results and documented antibiotic in the ED were selected. Only patients with appropriate antibiotic usage data were included in the final analysis.

### Data collection

Blood culture results and antibiotic usage data were obtained from electronic medical records (EMRs) (Supplementary Methods 1). The determination of whether a bacterial strain exhibited an extended spectrum beta-lactamase (ESBL) production or methicillin resistance (indicating methicillin-resistant *Staphylococcus aureus* (MRSA)) was based on the microbiological results. A strain was classified as having ESBL or MRSA resistance only if explicitly indicated in the microbiological findings. The criteria for identifying these resistance mechanisms were established by the Institute of Medical Microbiology. Survival data were requested from the Ministry of Interior, Deputy State Secretariat for Registries’ Management and supplemented with follow-up data from the EMR system. The national database has individual data on patient death status, including the date of death or the date of last status update (i.e. the date of last follow-up). The EMR has similar data for those who visited any of the university departments. Survival was evaluated from admission to the ED until the end of one-year follow-up, readmission, the patient’s death, or the end of the study, whichever happened sooner. Cases lost to one-year follow-up were right censored in the analysis. In these cases, the follow-up ended on the date of the last medical record in the hospital or on the date of readmission. Missing or incomplete data were noted in the figure and table legends.

Cases were classified as having resistance to ED-antibiotic therapy if at least one bacterial isolate demonstrated in vitro resistance to any administered antibiotic. Within this subgroup, therapy was classified as adequate if all detected bacterial isolates were susceptible to at least one of the administered antibiotics, or inadequate if at least one bacterium was resistant to all administered ED-antibiotics, based on in vitro testing (antibiogram). This classification aligns with the method described by Van Heuverswyn et al. [14], except for drugs not explicitly included in the susceptibility report. Cases in which not all administered antibiotics were covered by the antibiogram were classified as having unknown adequacy.

### Statistical analysis and visualization

Descriptive statistics were used to assess the distribution of variables. Continuous variables were summarized as means with standard deviations (SDs) or medians with interquartile ranges, while categorical variables were summarized as counts and percentages. Categorical data were compared with Pearson’s Chi-square test or Fisher’s exact test. Non-normally distributed data were analyzed with the Mann-Whitney U test. Log-rank tests were performed to establish an association between the predictor variables (species or antibiotic resistance) and survival. Multivariable Cox proportional hazard regression analysis was performed to calculate hazard ratios (HRs) with 95% confidence intervals (CIs), considering death as the primary event and adjusting for baseline risk factors (age, sex). Individuals were right-censored if they did not experience a death event by the end of the follow-up period or were lost to follow-up. Patients were censored at readmission even if they met the inclusion criteria. Data were collected and processed in Microsoft^®^ Excel^®^ (v2309). Statistical analyses were performed, and plots were created in IBM^®^ SPSS^®^ Statistics (v29). All statistical tests were two-tailed, and p-values of less than 0.05 were considered statistically significant. Figures were edited in Adobe^®^ Illustrator (v26).

## Results

### Population characteristics and overall mortality

**Fig. 1:**
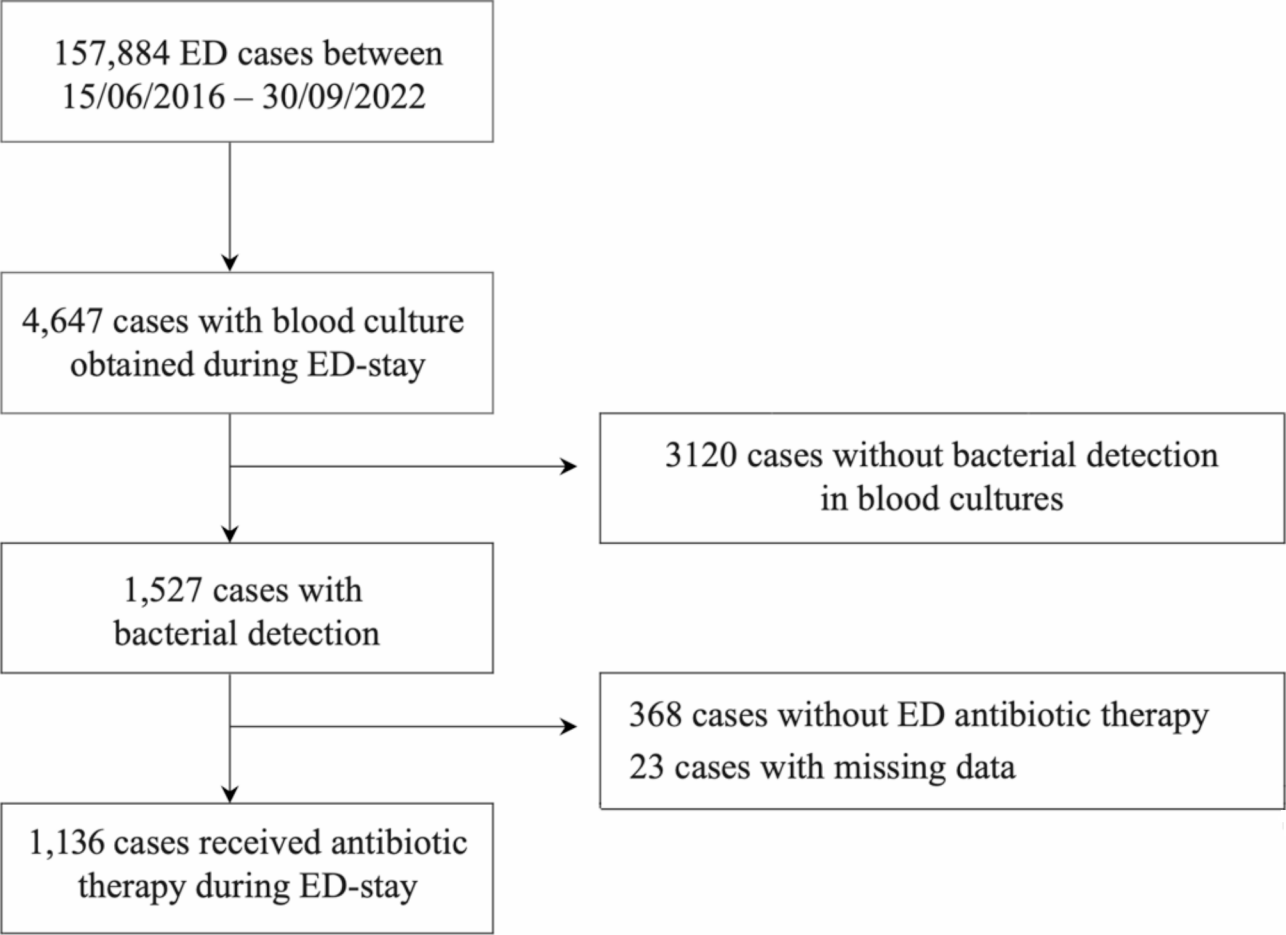
Patient selection flowchart. ED: emergency department

The number of visits to the ED between 15/06/2016 and 30/09/2022 was 157,884, of which blood cultures were obtained in 4,647 cases (2.94%). Blood cultures were positive in 1,527 cases (32.9% positivity rate). 1,159 patients received antibiotic therapy in the ED. Appropriate data on antibiotic usage were available in 1,136 cases that formed the study population (Fig. 1).

The three most common International Classification of Diseases (ICD) codes at discharge were related to sepsis (50.0%), urinary system disorders (19.6%), and pneumonia (19.5%) (Supplementary Table 1). One hundred ninety-four (17%) patients were admitted directly to the intensive care unit, and 46 patients (4%) died in the ED. Thirty-day and one-year mortality data were available for 1,122 and 1,085 cases, respectively. A total of 51 cases were lost to one-year follow-up, and they were right-censored in the analysis. The overall 30-day and one-year mortality rates were 38.6% and 61.8%, respectively (Table 1). Those who died were older.

### Microbiology and antibiotic resistance

**Fig. 2:**
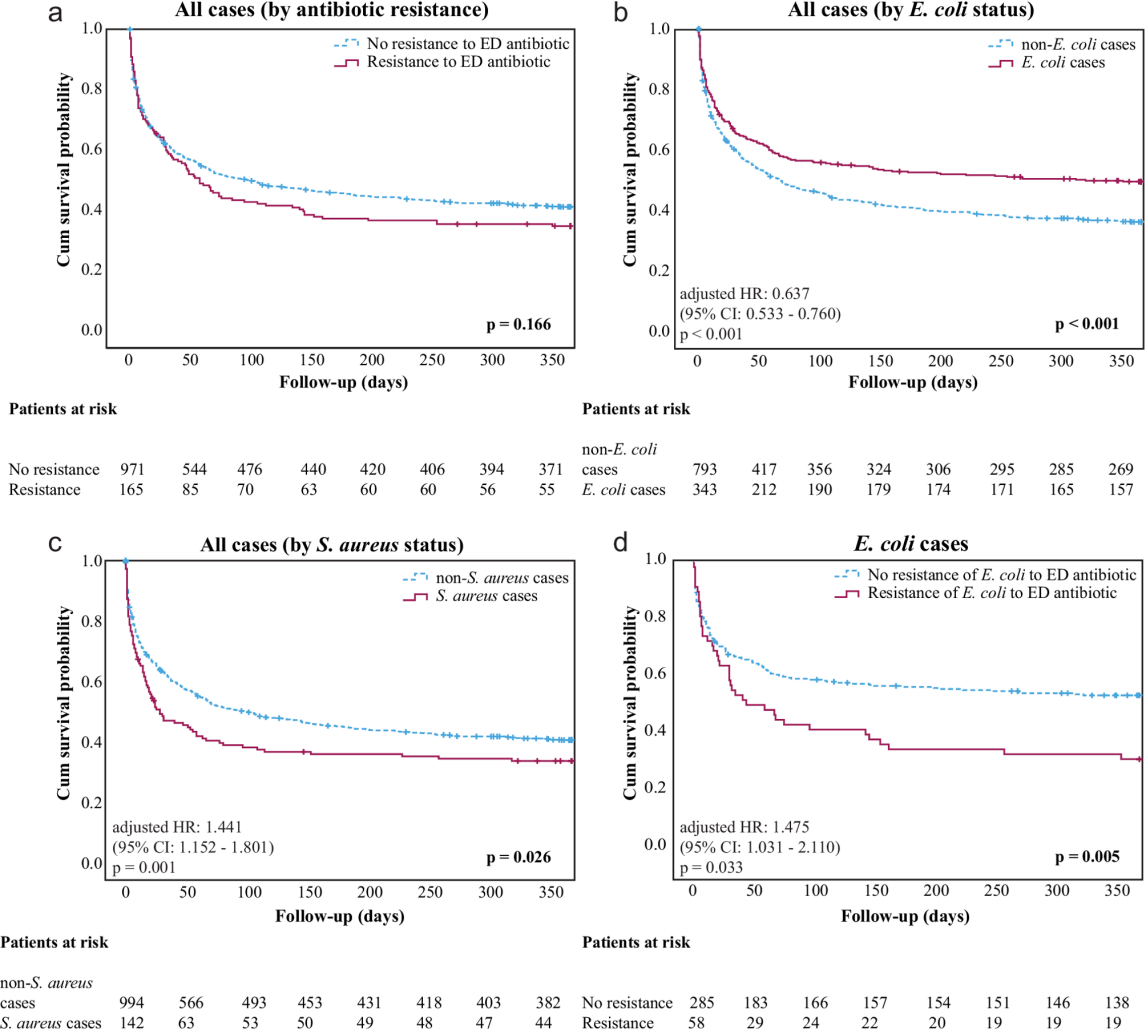
Kaplan-Meier curves of one-year survival. (**A**) Cum survival curves of cases with (solid line) and without (dashed line) resistance to the ED-administered antibiotic. (**B**) Cum survival curves of *E. coli*-positive (solid line) and *E. coli*-negative (dashed line) cases. (**C**) Cum survival curves of *S. aureus*-positive (solid line) and *S. aureus*-negative (dashed line) cases. (**D**) Cum survival curves of *E. coli*-positive cases with (solid line) and without (dashed line) resistance of *E. coli* to the ED-administered antibiotic. Step changes in the curves represent death. Cases were censored due to loss of follow-up or readmission with positive blood cultures and ED-initiated antibiotic therapy on the day of admission (vertical bars on Kaplan-Meier curves). For all panels, p-values of log-rank tests are shown on the bottom right; on the bottom left, adjusted hazard ratios are presented for multivariable Cox regression models controlled for age and sex. ED: emergency department; HR: hazard ratio; CI: confidence interval

The most frequently used antibiotics were ceftriaxone (*n* = 528), piperacillin/tazobactam (*n* = 233), clarithromycin (*n* = 181), and meropenem (*n* = 176) (Supplementary Table 2). Ceftriaxone was frequently used in combination with other antibiotics (57.8%), while piperacillin/tazobactam was mainly given as a single regimen (81.1%) (Supplementary Table 3). Resistance to at least one ED-administered antibiotic was found in 165 cases (14.5%) (Table 1). Even the combination therapy was inadequate in 84 cases (50.9%) (Supplementary Table 4). Twelve cases received adequate combination therapy, and in 69 cases, the adequacy could not be determined due to the absence of the administered antibiotics in the bacterial antibiogram. Among the cases with inadequate empirical antibiotic therapy, an ESBL strain was found in 45 cases (53.6%). The prevalence of resistance by antibiotic groups is shown in Supplementary Table 2. The prevalence of resistance was not different in those who died and those who survived in 30 days (66/433 (15.2%) vs. 98/689 (14.2), *p* = 0.701) or in one year (107/670 (16%) vs. 53/415 (12.8%), *p* = 0.175 (Table 1; Fig. 2a). This finding remained consistent after excluding cases with *Staphylococcus epidermidis* or *Staphylococcus hominis* positivity which are common contaminants [15] (Supplementary Fig. 1b). Patients who had resistance to at least one antibiotic, but still had adequate (combination) antibiotic therapy, had a tendency of lower mortality than those with inadequate therapy (5/12 (41.7%) vs. 55/81 (67.9%), *p* = 0.192 (pairwise log-rank)) (Supplementary Fig. 1a). A detailed list of all cases with resistance is provided in Supplementary Table 4.

**Table 1:** Characteristics of the cohort

Characteristics	All cases (*n* = 1,136)	30-day survival (*n* = 1,122)			one-year survival (*n* = 1,085)		
		Died (*n* = 433)	Survived (*n* = 689)	*p*-value	Died (*n* = 670)	Survived (*n* = 415)	*p*-value
**Demographic characteristics**							
Age [years] ^a^	72 (62–81)	74 (66–83)	70 (60–79)	**< 0.001**	74 (66–83)	68 (56–76)	**< 0.001**
Male ^b^	585 (51.5)	228 (52.7)	350 (50.8)	0.586	344 (51.3)	221 (53.3)	0.583
***Antibiotic resistance***							
Resistance to the given antibiotic ^b^	165 (14.5)	66 (15.2)	98 (14.2)	0.701	107 (16.0)	53 (12.8)	0.175

^a^ median (interquartile range); Mann-Whitney U test

^b^ n (%); Chi-squared test with continuity correction by Yates

Pathogen-specific resistance analysis showed that the most frequently resistant species (excluding *Staphylococcus hominis* and *Staphylococcus epidermidis*) was *Escherichia coli* (*E. coli*) (16.9% resistance against ED-administered antibiotics), with a high prevalence of ESBL strains (Table 2). Patients with *E. coli* infection who received ceftriaxone, ciprofloxacin, or piperacillin/tazobactam were resistantin 19.6%, 36.4%, and 25.6% of cases, respectively (Table 2). The resistant strains were ESBL-producing in 84.5%. *K. pneumoniae* was resistant to ED-administered antibiotics in 10 cases (13.3%), 9 of which were ESBL (Table 2, Supplementary Table 5). *Staphylococcus aureus* (*S. aureus*) was found in 142 cases, showing an overall antibiotic resistance of 5.6%. However, the three most commonly used antibiotics (ceftriaxone, meropenem, and piperacillin/tazobactam) were not included in the *S. aureus* antibiogram. Among the given antibiotics that were later tested for susceptibility, amoxicillin/clavulanic acid had the highest resistance rate, followed by clarithromycin and azithromycin (25%, 23.8%, and 7.7%), respectively. The MRSA rate among resistant strains against ED-initiated antibiotic therapy was 37.5% (Table 2). A complete list of species resistant to at least one given antibiotic is provided in Supplementary Table 5.

**Table 2:** The most common resistant pathogens and their antibiotic resistance. Complete data are provided in supplementary table 5

		*Escherichia coli* (*n* = 343)	*Klebsiella pneumoniae* (*n* = 75)	*Pseudomonas aeruginosa* (*n* = 40)	*Staphylococcus aureus* (*n* = 142)	*Staphylococcus epidermidis* (*n* = 124)	*Staphylococcus hominis* (*n* = 117)	*Streptococcus pneumoniae* (*n* = 59)
**AB-specific resistance**	**Amoxicillin / clavulanic acid**	3/5 (60%) *ESBL: 0*	0/4 (0%)	N.A./2	2/8 (25%) *MRSA: 2*	4/6 (66.7%)	1/3 (33.3%)	-
	**Azithromycin**	N.A./13	N.A./2	N.A./1	1/13 (7.7%) *MRSA: 1*	5/9 (55.6%)	5/13 (38.5%)	0/4 (0%)
	**Ceftriaxone**	30/153 (19.6%) *ESBL: 29*	6/30 (20%) *ESBL: 6*	N.A./10	N.A./60	N.A./60	N.A./65	0/44 (0%)
	**Ciprofloxacin**	4/11 (36.4%) *ESBL: 2*	2/3 (66.7%) *ESBL: 1*	-	0/1 (0%)	2/2 (100%)	1/3 (33.3%)	-
	**Clarithromycin**	N.A./31	N.A./6	N.A./2	5/21 (23.8%) *MRSA: 0*	15/29 (51.7%)	16/22 (72.7%)	2/31 (6.5%)
	**Clindamycin**	N.A./9	N.A./1	N.A./1	0/6 (0%)	2/4 (50%)	2/5 (40%)	0/2 (0%)
	**Metronidazole**	N.A./17	N.A./2	N.A./1	-	N.A./4	N.A./3	-
	**Moxifloxacin**	0/3 (0%)	0/1 (0%)	N.A./1	0/1 (0%)	3/4 (75%)	-	0/3 (0%)
	**Piperacillin/** **Tazobactam**	20/78 (25.6%) *ESBL: 17*	1/10 (10%) *ESBL: 1*	4/12 (33.3%)	N.A./27	N.A./28	N.A./21	0/7 (0%)
**Overall resistance** *ESBL or MRSA strain*		58 (16.9%) *49 (84.5%)*	10 (13.3%) *9 (90%)*	5 (12.5%) -	8 (5.6%) *3 (37.5%)*	33 (26.6%) -	27 (23.1%) -	2 (3.4%) -

Nominator = number of cases with bacterial resistance against the given antibiotic; Denominator = total number of cases with bacterial presence receiving the defined antibiotic; N.A. = antibiotic was not part of the antibiogram of this bacterium

The number of ESBL and MRSA cases resistant to the administered antibiotics are shown in *italic*. Resistance to at least one given antibiotic are shown

AB = antibiotic

### Pathogen-specific mortality

Patients with *E. coli* had a lower one-year mortality rate than patients with *E. coli*-negative infection (52.9% vs. 65.5%, *p* < 0.001), although *E. coli* cases were older and had a higher rate of antibiotic resistance (Table 3). Thirty-day mortality was also lower by 5.7% in the *E. coli* group, although this difference was not significant (*p* = 0.081). Conversely, patients with *S. aureus* had higher 30-day mortality compared to *S. aureus-*negative patients (51.8% vs. 36.7%, *p* < 0.001) (Table 3). In a Cox proportional multivariable regression model adjusting for age and sex, *E. coli* positivity was associated with a 36.3% lower risk of death in one year (HR: 0.637, 95% CI 0.533–0.760, *p* < 0.001) (Fig. 2b). In a similar model, *S. aureus* positivity was associated with a 44.1% higher risk of death in one year (HR: 1.441, 95% CI 1.152–1.801, *p* = 0.001) (Fig. 2c).

When infection source (based on ICD codes) was included in the multivariable models, *E. coli* positivity remained associated with a 33.2% lower risk of death within one year (HR: 0.668; 95% CI 0.557-0.800; *p* < 0.001) (Supplementary Table 7). The presence of ICD code N39 (urinary tract infections) was independently associated with a reduced risk of death within one year (HR: 0.697; 95% CI 0.569–0.855; *p* < 0.001).

For *S. aureus* positivity, N39 was similarly associated with a lower risk of death (HR: 0.665; 95% CI 0.543–0.814; *p* < 0.001). The proportion of cases coded as A41 (sepsis) was higher in the *S. aureus*-positive group compared to the *S. aureus-*negative group (64.8% vs. 47.9%, Supplementary Table 6). When ICD code A41 was added to the regression model, it was found that this code was independently associated with a higher risk of death in one year (HR: 1.234, 95% CI 1.053–1.447, *p* = 0.010), while *S. aureus* positivity remained associated. Other bacterial positivity (*Klebsiella pneumoniae*, *Staphylococcus hominis*, or *Staphylococcus epidermidis*) were not associated with survival (Supplementary Fig. 2).

**Table 3:** *E. Coli* and *S. Aureus* bacteremia. Baseline characteristics, antibiotic resistance and survival depending on *E. Coli* and *S. Aureus* positivity. Subgroup analysis of *E. Coli* and *S. Aureus* positive cases

Characteristics	All patients (*n* = 1,136)							*E. coli* subgroup (*n* = 343)			*S. aureus* subgroup (*n* = 142)		
	Total	By *E. coli* positivity			By *S. aureus* positivity								
		*E. coli*- positive (*n* = 343)	*E. coli*- negative (*n* = 793)	*p*	*S. aureus*- positive (*n* = 142)	*S. aureus*- negative (*n* = 994)	*p*	Resistance^$^ (*n* = 58)	No resistance^$^ (*n* = 285)	*p*	Resistance^$^ (*n* = 8)	No resistance^$^ (*n* = 134)	*p*
Age, years ^a^	72 (62–81)	75 (66–82)	70 (61–79)	**< 0.001**	69 (61–77)	72 (62–81)	0.059	77 (69–84)	74 (66–82)	0.204	71 (65–79)	69 (60–77)	0.556
Male sex ^b^	585 (51.5)	130 (37.9)	455 (57.4)	**< 0.001**	87 (61.3)	498 (50.1)	**0.016**	26 (44.8)	104 (36.5)	0.296	6 (75)	81 (60.4)	0.485
AB resistance ^b, *^	165 (14.5)	65 (19.0)	100 (12.6)	**0.007**	12 (8.5)	153 (15.4)	**0.039**	-	-	-	-	-	-
Died in 30 days ^b,£^	433 (38.6)	118 (34.6)	315 (40.3)	0.081	72 (51.8)	361 (36.7)	**< 0.001**	25 (43.1)	93 (32.9)	0.180	4 (50)	68 (51.9)	1.000
Died in one year ^b,#^	670 (61.8)	172 (52.9)	498 (65.5)	**< 0.001**	92 (68.7)	578 (60.8)	0.097	40 (69.0)	132 (49.4)	**0.011**	5 (71.4)	87 (68.5)	1.000

^a^ median (interquartile range); Mann-Whitney’s U test

^b^ n (%); Chi-squared test with continuity correction by Yates or Fisher’s exact test when expected cell count of ≥ 1 cells was less than 5

^*^ resistance of ≥ 1 pathogen to ≥ 1 emergency department-initiated antibiotic therapy

^$^ resistance/ no resistance of *S. aureus* against ≥ 1 emergency department-initiated antibiotic therapy

^£^ data available for 1122 cases, 341 *E. coli* cases, 139 *S. aureus* cases

^#^ data available for 1085 cases, 325 *E. coli* cases, 136 *S. aureus* cases

AB = antibiotic.

In patients with *E. coli* infection, resistance of *E. coli* to ED-administered antibiotics was associated with a 10.2% higher 30-day and 19.6% higher one-year mortality (*p* = 0.180 and *p* = 0.011), respectively (Table 3). In the multivariable Cox model (adjusted for age and sex), antibiotic resistance of *E. coli* was associated with a 47.5% increased risk of death at one year (HR: 1.475, 95% CI 1.031–2.110, *p* = 0.033) (Fig. 2d). This association of antibiotic resistance with survival was not present in other bacterial subgroups (*Klebsiella pneumoniae*, *S. aureus*, *Staphylococcus epidermidis*, and *Staphylococcus hominis*) (Supplementary Fig. 1c-f).

## Discussion

Sepsis is a high-mortality condition necessitating early empiric antibiotic therapy [4, 16]. While previous studies suggest that inadequate initial empiric antibiotic therapy is associated with higher mortality [17, 18], we found no association between resistance to initial antibiotic therapy with short- or long-term overall mortality. However, a subgroup of patients with *E. coli* infection showed an association of antibiotic resistance with mortality. We also found associations between specific pathogens and mortality (in the case of *E. coli* and *S. aureus*), which align with previous findings [19, 20].

Our findings revealed a notably high 30-day mortality rate of 38.6%, compared to the previously observed 28-day mortality rates of 9.25% and 14% in studies conducted in the ED [21, 22]. On one hand, our patient cohort likely represented an older population, as it included older patients than the population studied by Lee et al. (mean age: 64.6 years) and the patients included by Rannikko et al. (median age: 68 years). On the other hand, our cohort was likely sicker. Even though Rannikko et al. focused on patients with blood culture-positive sepsis, our cohort had the highest intensive care unit admission rate of 17%, compared to the 10% reported by Rannikko et al. and the 12.6% by Lee et al., who, similarly to our inclusion criteria, included patients with positive blood cultures regardless of whether they were septic or not. Our inclusion of a sicker and older population is supported by 2021 data from the World Health Organization (WHO). In 2021, Hungary’s life expectancy was 74.4 years, with a healthy life expectancy (HALE) of 64.8 years, both lower than the reported European averages (life expectancy: 76.3 years; HALE: 66 years). This reflects poorer overall health in Hungary, partially explaining the higher mortality rate in our cohort [23, 24].

The low frequency of blood culture draws (2.9%) compared to previous studies [21, 25] and the high positive blood culture rate (32.9%) suggest that during the study period blood culture was used in higher-risk patients and not as a screening tool. In our ED, blood cultures are primarily drawn from patients with suspected sepsis or septic shock, which is supported by ICD coding.

While ICD coding has limitations in diagnostic precision, the fact that 50% of patients had the A41 ICD code (sepsis) suggests that sepsis or septic shock was likely suspected in at least half of these patients. This selection of a likely sicker population, and the potential underrepresentation of less severe cases might have contributed to the higher blood culture positivity and mortality rate observed. The difference in blood culture positivity rates likely reflects differences in sampling strategies, as Kao et al. included all febrile patients regardless of clinical severity, resulting in a positivity rate of only 13.5% [25], whereas in our ED blood cultures are not drawn from every febrile patient.

Remarkably, resistant *E. coli* strains were ESBL-producing in more than 4 out of 5 cases. Moreover, patients with *E. coli* infection who received piperacillin/tazobactam had resistance to it in 25.6% of cases. This finding supports the need for careful consideration of using carbapenems over piperacillin/tazobactam in patients with *E. coli* or *K. pneumoniae* bloodstream infections if ESBL is suspected [26]. However, antimicrobial stewardship principles should guide therapy selection, considering the infection site, patient characteristics, and local resistance patterns, as alternatives to carbapenems may be appropriate in certain clinical settings.

The European Centre for Disease Prevention and Control (ECDC) Surveillance Atlas of Infectious Disease Data shows that in Hungary in 2021, the highest resistance in *E. coli* cases was for aminopenicillins (58.5%), fluoroquinolones (28.1%), third-generation cephalosporins (20.4%), and aminoglycosides (17.5%) [27]. In recent years, *E. coli* resistance rates have increased for all antibiotics except aminopenicillins [28]. The resistance rates reported in our study are comparable to the nationwide antibiotic resistance data reported by ECDC [28, 29]. Our data are based on an ED setting and, therefore, likely included patients with community-acquired infections. Previous studies showed that pathogen distributions and resistance patterns differ between hospital-acquired and community-acquired infections [30, 31].

Our study has several strengths and limitations. The strengths are that this study (1) focuses on a large cohort of ED patients in the understudied Central-Eastern European area and (2) provides high-quality long-term follow-up data. Limitations of the study include (1) its retrospective nature and single-center study design; (2) only blood cultures drawn in the ED were considered for inclusion/exclusion, thus patients with false-negative blood cultures might have been excluded; (3) patients who did not receive ED empiric antibiotic therapy were excluded, leading to a smaller sample size; (4) antibiotic resistance profiles may vary from hospital to hospital, which limits the generalizability of our findings; (5) only patients admitted to the ED were included, which further limits generalizability of the results; (6) time to first antibiotic therapy was not examined in this study, but it is a contributing factor to mortality in patients with septic shock [3]; (7) due to the lack of data, differentiation between deaths due to hospitalization-related factors and those due to pre-existing conditions or external causes was not possible; (8) adjustments of antibiotic therapy were not available in our dataset, which might also have impacted mortality outcomes. (9) While our study suggests that the population was sicker based on patients’ age and intensive care unit admission rates, the lack of severity indices in the dataset represents a limitation, as such indices would have allowed a more comprehensive comparison of patient health across populations.

## Conclusion

Blood culture-positive ED patients had a high rate of mortality (30-day: 38.6%, one-year: 61.8%). While overall mortality was not associated with resistance to empiric ED antibiotic therapy, it was linked to a 1.5-fold increased risk of death in one year, in patients who had resistant *E. coli* infection. Antibiotic resistance and the high proportion of ESBL strains in the emergency department are worrying. Specific subgroups of patients with bacteremia are likely to benefit from early adequate antibiotic therapy.

## Electronic supplementary material

Below is the link to the electronic supplementary material.

Supplementary Material 1: Supplementary Methods 1. Supplementary Table 1 Most commonly used ICD codes (n = 1,136). Supplementary Table 2 Antibiotic use and resistance. Prevalence of antibiotic resistance based on blood cultures sampled in the emergency department in the entire cohort. If the antibiotic was not part of the antibiogram, it was categorized as ‘non-resistant’. Supplementary Table 3 Initial empiric antibiotic therapy at the ED. Supplementary Table 4 Antibiotic use and identified pathogens in cases with resistance to ED antibiotic therapy (n = 165).

Supplementary Material 2: Supplementary Table 5 Identified species with resistance to at least one ED antibiotic. Supplementary Table 6. Overview of all utilized ICD codes (n = 1,136) with a comparative analysis between E. Coli-positive and non-E. Coli-positive cases and between S. Aureus-positive and non-S. Aureus-positive cases. Supplementary Table 7 Cox proportional hazards multivariable regression models for S. aureus bacteremia and mortality adjusted for A) Age, Sex, ICD Codes: U07, J18, N39 B) Age, Sex, ICD Codes: A41, U07, J18, N39. Cox proportional hazards multivariable regression models for E. coli bacteremia and mortality adjusted for C) Age, Sex, ICD Codes: U07, J18, N39 D) Age, Sex, ICD Codes: A41, U07, J18, N39

Supplementary Material 3: Supplementary Fig. 1 Kaplan-Meier curves of 1-year survival. A) Cum survival curves of cases with non adequate antibiotic therapy (n = 84, red line), with unknown adequacy of antibiotic therapy (n = 69, turquoise line) and with adequate antibiotic combination therapy (n = 12, black line) B) Cum survival curves of cases after exclusion of *S. epidermidis* and *S. hominis* with (n = 109, solid line) and without (n = 814) bacteria resistant to an ED antibiotic. C) Cum survival curves of *S. hominis* cases with (n = 27) and without (n = 90) resistance of *S. hominis* to an ED antibiotic. D) Cum survival curves of *K. pneumoniae* cases with (n = 10) and without (n = 65) resistance of *K. pneumoniae* to an ED antibiotic. E) Cum survival curves of *S. aureus* cases with (n = 8) and without (n = 134) resistance of *S. aureus* to an ED antibiotic. F) Cum survival curves of *S. epidermidis* cases with (n = 33) and without (n = 91) resistance of *S. epidermidis* to an ED antibiotic. For all panels, the follow-up period was one year; p-values of log-rank tests are shown

Supplementary Material 4: Supplementary Fig. 2 Kaplan-Meier curves of 1-year survival. A) Cum survival curves of *S. hominis*-positive (n = 117) and *S. hominis*-negative (n = 1,019) cases. B) Cum survival curves of *K. pneumoniae*-positive (n = 75) and *K. pneumoniae*-negative (n = 1,061) cases. C) Cum survival curves of *S. epidermidis*-positive (n = 124) and *S. epidermidis*-negative (n = 1,012). For all panels, the follow-up period was one year; p-values of log-rank tests are shown

## Data Availability

All data analyzed during this study are included in the supplementary information files or are available at the corresponding author upon reasonable request.

## Abbreviations

AB: Antibiotic
CI: Confidence interval
ECDC: European Centre for Disease Prevention and Control
ED: Emergency department
EMR: Electronic medical record
ESBL: Extended spectrum beta-lactamase
HALE: Healthy life expectancy
HR: Hazard ratio
ICD: International Classification of Diseases
MRSA: Methicillin-resistant Staphylococcus aureus
SD: Standard deviation
WHO: World Health Organization
