# Supplementary material for: Empiric antibiotic therapy resistance and mortality in emergency department patients with bloodstream infection: a retrospective cohort study
Source: BMC Emerg Med. 2025 Jan 27;25:18. doi: 10.1186/s12873-025-01177-0 (PMC11773878; doi:10.1186/s12873-025-01177-0)
Supplement: Supplementary file 1 — Supplementary Material 1: Supplementary Methods 1. Supplementary Table 1 Most commonly used ICD codes (n = 1,136). Supplementary Table 2 Antibiotic use and resistance. Prevalence of antibiotic resistance based on blood cultures sampled in the emergency department in the entire cohort. If the antibiotic was not part of the antibiogram, it was categorized as ‘non-resistant’. Supplementary Table 3 Initial empiric antibiotic therapy at the ED. Supplementary Table 4 Antibiotic use and identified pathogens in cases with resistance to ED antibiotic therapy (n = 165). [file 12873_2025_1177_MOESM1_ESM.docx]

| ***ICD code groups**** | n (% of 1,136) |
| --- | --- |
| ***Infections/ Sepsis*** | |
| A41 Other sepsis | 568 (50.0%) |
| U07 Emergency use code (COVID-19) | 107 (9.4%) |
| ***Blood and blood-forming*** | |
| D64 Other anaemias | 40 (3.5%) |
| ***Endocrine, nutritional, and metabolic diseases*** | |
| E11 Type 2 diabetes mellitus | 24 (2.1%) |
| E86 Volume depletion | 211 (18.6%) |
| ***Circulatory system*** | |
| I10 Essential (primary) hypertension | 38 (3.3%) |
| I48 Atrial fibrillation and flutter | 30 (2.6%) |
| I50 Heart failure | 58 (5.1%) |
| ***Respiratory system*** | |
| J15 Bacterial pneumonia, not elsewhere classified | 26 (2.3%) |
| J18 Pneumonia, organism unspecified | 221 (19.5%) |
| J44 Other chronic obstructive pulmonary disease | 32 (2.8%) |
| J96 Respiratory failure, not elsewhere classified | 152 (13.4%) |
| ***Urinary system*** | |
| N18 Chronic kidney disease | 27 (2.4%) |
| N30 Cystitis | 27 (2.4%) |
| N39 Other disorders of urinary system | 223 (19.6%) |
| ***Symptoms, signs, and abnormal clinical laboratory findings*** | |
| R06 Abnormalities of breathing | 80 (7.0%) |
| R10 Abdominal and pelvic pain | 100 (8.8%) |
| R41 Other symptoms and signs involving cognitive functions and awareness | 25 (2.2%) |
| R50 Fever of other and unknown origin | 113 (9.9%) |
| *for case-coding according to <https://icd.who.int/browse10/2019/en#/> | |

**Supplementary Table 1** Most commonly used ICD codes (n = 1,136). A complete list of all utilized ICD codes is provided in supplementary table 6.

**Supplementary Table 2** Antibiotic use and resistance. Prevalence of antibiotic resistance based on blood cultures sampled in the emergency department in the entire cohort. If the antibiotic was not part of the antibiogram, it was categorized as 'non-resistant’.

|  | **ED antibiotic therapy** | | **Prevalence of antibiotic resistance in the full cohort**  **(% of 1,136)** |
| --- | --- | --- | --- |
|  | **Antibiotic administration** | **Resistance**  **against**  **ED antibiotic therapy** |  |
| **Total** | **1,136 (100%)** | **165 (14%)** | **-** |
| Ceftriaxone | 528 (46.5%) | 41 (7.77%) | 93 (8.2%) |
| Piperacillin/Tazobactam | 233 (20.5%) | 25 (10.7%) | 119 (10.5%) |
| Clarithromycin | 181 (15.9%) | 43 (23.8%) | 235 (20.7%) |
| Meropenem | 176 (15.5%) | 1 (0.6%) | 9 (0.8%) |
| Imipenem/Cilastatin | 100 (8.8%) | 0 | 15 (1.3%) |
| Vancomycin | 81 (7.1%) | 0 | 4 (0.4%) |
| Azithromycin | 69 (6.1%) | 14 (20.3%) | 235 (20.7%) |
| Metronidazole | 41 (3.6%) | 2 (4.9%) | 12 (1.1%) |
| Clindamycin | 40 (3.5%) | 6 (15%) | 175 (15.4%) |
| Amoxicillin/ clavulanic acid | 35 (3.1%) | 14 (40%) | 271 (23.9%) |
| Ciprofloxacin | 25 (2.2%) | 10 (40%) | 262 (23.1%) |
| Ampicillin/sulbactam | 20 (1.8%) | 1 (5%) | 147 (12.9%) |
| Gentamicin | 18 (1.6%) | 1 (5.6%) | 130 (11.4%) |
| Moxifloxacin | 13 (1.1%) | 4 (30.8%) | 258 (22.7) |
| Amikacin | 7 (0.6%) | 2 (28.6%) | 185 (16.3%) |
| Penicillin | 5 (0.4%) | 0 | 49 (4.3%) |
| Erythromycin | 3 (0.3%) | 1 (33.33%) | 236 (20.8%) |
| Levofloxacin | 2 (0.2%) | 2 (100%) | 264 (23.2%) |
| Cefazolin | 1 (0.1%) | 0 | 130 (11.4%) |
| Cefuroxim | 1 (0.1%) | 1 (100%) | 195 (17.2%) |

| **Antibiotic therapy** | **n (% of 1,136)** |
| --- | --- |
| Ceftriaxone monotherapy | 223 (19.6%) |
| Piperacillin/ tazobactam monotherapy | 189 (16.6%) |
| Ceftriaxone + Clarithromycin | 159 (14.0%) |
| Meropenem monotherapy | 143 (12.6%) |
| Imipenem cilastatine | 83 (7.3 %) |
| Ceftriaxone + Azithromycine | 61 (5.4%) |
| Amoxicillin clavulanic acid | 27 (2.4%) |
| Ceftriaxone + Metronidazol | 18 (1.6%) |
| Piperacillin/ Tazobactam + Vancomycin | 14 (1.2%) |
| Ciprofloxacin monotherapy | 14 (1.2%) |
| Ceftriaxone + Clindamycin | 12 (1.1%) |
| Clindamycin monotherapy | 12 (1.1%) |
| Other | 181 (15.9%) |

**Supplementary Table 3** Initial empiric antibiotic therapy at the ED.

**Supplementary Table 4** Antibiotic use and identified pathogens in cases with resistance to ED antibiotic therapy (n = 165).

| **Antibiotic therapy** | **n** | **Bacteria grown in blood cultures with therapy-related antibiogram** |
| --- | --- | --- |
| Amoxicillin/clavulanic acid | 3 | *Staphylococcus epidermidis* (Amoxicillin/clavulanic acid:R) |
|  | 2 | *Staphylococcus aureus* (Amoxicillin/clavulanic acid:R) (MRSA) |
|  | 1 | *Escherichia coli* (Amoxicillin/clavulanic acid:R) |
|  |  | *Klebsiella pneumoniae* (Amoxicillin/clavulanic acid:S) |
|  | 2 | *Escherichia coli* (Amoxicillin/clavulanic acid:R) |
|  | 1 | *Morganella morganii* (Amoxicillin/clavulanic acid:R) |
|  |  | *Pseudomonas aeruginosa* |
|  | 1 | *Staphylococcus hominis (Amoxicillin/clavulanic acid:R)* |
| Azithromycin | 1 | *Staphylococcus hominis* (Azithromycin:R) |
| Ceftriaxone | 18 | *Escherichia coli* (Ceftriaxone:R) (ESBL) |
|  | 1 | *Escherichia coli* (Ceftriaxone:R) |
|  | 1 | *Escherichia coli* (Ceftriaxone:R) (ESBL) |
|  |  | *Cutibacterium acnes* |
|  | 1 | *Escherichia coli* (Ceftriaxone:R) (ESBL) |
|  |  | *Staphylococcus epidermidis* |
|  | 1 | *Escherichia coli* (Ceftriaxone:R) (ESBL) |
|  |  | *Klebsiella pneumoniae* (Ceftriaxone:S) |
|  | 1 | *Klebsiella aerogenes* (Ceftriaxone:S) |
|  |  | *Klebsiella aerogenes* (Ceftriaxone:R) |
|  | 1 | *Klebsiella pneumoniae* (Ceftriaxone:R) (ESBL) |
|  | 1 | *Klebsiella pneumoniae* (Ceftriaxone:R) (ESBL) |
|  |  | *Proteus vulgaris* (Ceftriaxone:S) |
|  | 1 | *Klebsiella pneumoniae* (Ceftriaxone:R) (ESBL) |
|  |  | *Staphylococcus hominis* |
|  | 1 | *Paenibacillus sp.* (Ceftriaxone:R) |
|  | 1 | *Pseudomonas aeruginosa* |
|  |  | *Enterobacter cloacae* (Ceftriaxone:R) |
|  | 1 | *Staphylococcus aureus* |
|  |  | *Klebsiella pneumoniae* (Ceftriaxone:R) (ESBL) |
|  | 1 | *Trueperella bernardiae* |
|  |  | *Escherichia coli* (Ceftriaxone:R) (ESBL) |
|  |  | *Proteus mirabilis* (Ceftriaxone:S) |
|  |  | *Morganella morganii* (Ceftriaxone:S) |
| Ceftriaxone, Ampicillin/sulbactam | 1 | *Escherichia coli* (Ampicillin/sulbactam:R, Ceftriaxone:S) |
| Ceftriaxone, Azithromycin | 4 | *Staphylococcus hominis* (Azithromycin:R) |
|  | 3 | *Staphylococcus epidermidis* (Azithromycin:R) |
|  | 1 | *Staphylococcus pettenkoferi* (Azithromycin:R) |
|  | 1 | *Escherichia coli* (Ceftriaxone:R) (ESBL) |
|  | 1 | *Klebsiella pneumoniae* (Ceftriaxone:R) (ESBL) |
|  | 1 | *Staphylococcus aureus* (Azithromycin:R) (MRSA) |
|  | 1 | *Staphylococcus caprae* (Azithromycin:R) |
|  | 1 | *Staphylococcus epidermidis* (Azithromycin:R) |
|  |  | *Corynebacterium imitans* (Azithromycin:R) |
|  |  | *Aerococcus viridans* (Ceftriaxone:S) |
|  | 1 | *Staphylococcus epidermidis* (Azithromycin:R) |
|  |  | *Staphylococcus aureus* (Azithromycin:S) |
| Ceftriaxone, Ciprofloxacin | 1 | *Staphylococcus hominis* (Ciprofloxacin:R) |
|  |  | *Staphylococcus epidermidis* (Ciprofloxacin:R) |
| Ceftriaxone, Clarithromycin | 11 | *Staphylococcus hominis* (Clarithromycin:R) |
|  | 10 | *Staphylococcus epidermidis* (Clarithromycin:R) |
|  | 5 | *Staphylococcus aureus* (Clarithromycin:R) |
|  | 3 | *Escherichia coli* (Ceftriaxone:R) (ESBL) |
|  | 2 | *Streptococcus pneumoniae* (Ceftriaxone:S, Clarithromycin:R) |
|  | 1 | *Enterococcus faecium* (Vancomycin resistent strain) |
|  |  | *Staphylococcus hominis* (Clarithromycin:R) |
|  | 1 | *Escherichia coli* (Ceftriaxone:S) |
|  |  | *Staphylococcus epidermidis* (Clarithromycin:R) |
|  | 1 | *Escherichia coli* (Ceftriaxone:S) |
|  |  | *Staphylococcus hominis* (Clarithromycin:R) |
|  | 1 | *Escherichia coli* (Ceftriaxone:R) (ESBL) |
|  |  | *Staphylococcus simulans* (Clarithromycin:R) |
|  | 1 | *Lactobacillus sp.* |
|  |  | *Dietzia sp.* (Ceftriaxone:R, Clarithromycin:S) |
|  |  | *Staphylococcus hominis* (Clarithromycin:R) |
|  | 1 | *Staphylococcus capitis* (Clarithromycin:R) |
|  |  | *Streptococcus salivarius* (Ceftriaxone:S) |
|  | 1 | *Staphylococcus caprae* (Clarithromycin:R) |
|  | 1 | *Staphylococcus epidermidis* (Clarithromycin:R) |
|  |  | *Staphylococcus pettenkoferi* (Clarithromycin:S) |
|  |  | *Staphylococcus aureus* (Clarithromycin:S) |
|  | 1 | *Staphylococcus epidermidis* (Clarithromycin:R) |
|  |  | *Streptococcus pneumoniae* (Ceftriaxone:S, Clarithromycin:S) |
|  | 1 | *Staphylococcus haemolyticus* (Clarithromycin:R) |
|  |  | *Staphylococcus hominis* (Clarithromycin:R) |
|  | 1 | *Staphylococcus warneri* (Clarithromycin:R) |
|  | 1 | *Streptococcus dysgalactiae* (Ceftriaxone:S, Clarithromycin:R) |
| Ceftriaxone, Clarithromycin, Clindamycin | 1 | *Staphylococcus epidermidis* (Clarithromycin:R, Clindamycin:S) |
| Ceftriaxone, Clarithromycin, Metronidazole | 1 | *Klebsiella aerogenes* (Ceftriaxone:R) |
|  |  | *Enterococcus casseliflavus* |
|  |  | *Escherichia coli* (Ceftriaxone:S) |
| Ceftriaxone, Clindamycin | 2 | *Escherichia coli* (Ceftriaxone:R) (ESBL) |
| Ceftriaxon, Clindamycin, Amoxicillin/clavulanic acid | 1 | *Staphylococcus xylosus* (Amoxicillin/clavulanic acid:S, Clindamycin:R) |
| Ceftriaxone, Metronidazole | 1 | *Klebsiella pneumoniae* (Ceftriaxone:R) (ESBL) |
| Ceftriaxone, Vancomycin, Amoxicillin/clavulanic acid | 1 | *Escherichia coli* (Amoxicillin/clavulanic acid:S, Ceftriaxone:S) |
|  |  | *Staphylococcus epidermidis* (Amoxicillin/clavulanic acid:R, Vancomycin:S) |
|  |  | *Klebsiella pneumoniae* (Amoxicillin/clavulanic acid:S, Ceftriaxone:S) |
| Cefuroxime | 1 | *Staphylococcus hominis* (Cefuroxime:R) |
| Ciprofloxacin | 2 | *Escherichia coli* (Ciprofloxacin:R) |
|  | 2 | *Escherichia coli* (Ciprofloxacin:R) (ESBL) |
|  | 1 | *Clostridium perfringens* |
|  |  | *Escherichia coli* (Ciprofloxacin:S) |
|  |  | *Staphylococcus capitis* (Ciprofloxacin:R) |
|  |  | *Streptococcus gallolyticus* |
|  | 1 | *Klebsiella pneumoniae* (Ciprofloxacin:R) |
|  | 1 | *Staphylococcus epidermidis* (Ciprofloxacin:R) |
| Clarithromycin | 1 | *Staphylococcus epidermidis* (Clarithromycin:R) |
|  | 1 | *Staphylococcus hominis* (Clarithromycin:R) |
| Clarithromycin, Amoxicillin/clavulanic acid | 1 | *Corynebacterium mucifaciens* (Amoxicillin/clavulanic acid:R) |
| Clindamycin | 1 | *Staphylococcus capitis* (Clindamycin:S) |
|  |  | *Staphylococcus hominis* (Clindamycin:S) |
|  |  | *Staphylococcus epidermidis* (Clindamycin:R) |
|  | 1 | *Staphylococcus epidermidis* (Clindamycin:R) |
|  | 1 | *Staphylococcus hominis* (Clindamycin:R) |
|  | 1 | *Streptococcus agalactiae* (Clindamycin:R) |
| Imipenem/cilastatin, Ciprofloxacin | 1 | *Klebsiella pneumoniae* (Imipenem:S, Ciprofloxacin:R) (ESBL) |
| Imipenem/cilastatin, Metronidazole | 1 | *Staphylococcus capitis* |
|  |  | *Propionibacterium acnes* (Imipenem:S, Metronidazol:R) |
| Levofloxacin | 1 | *Staphylococcus hominis* (Levofloxacin:R) |
|  | 1 | *Klebsiella pneumoniae* (Levofloxacin:R) (ESBL) |
| Meropenem | 1 | *Pseudomonas aeruginosa* (Meropenem:R) |
| Metronidazole, Ciprofloxacin | 1 | Campylobacter jejuni (Ciprofloxacin:R) |
|  | 1 | *Staphylococcus hominis* (Ciprofloxacin:S) |
|  |  | *Propionibacterium acnes* (Metronidazole:R) |
| Metronidazole, Moxifloxacin | 1 | *Klebsiella oxytoca* (Moxifloxacin:R) |
|  |  | *Pseudomonas aeruginosa* |
| Moxifloxacin | 1 | *Corynebacterium striatum* |
|  |  | *Klebsiella pneumoniae* (Moxifloxacin:S) |
|  |  | *Staphylococcus epidermidis* (Moxifloxacin:R) |
|  | 1 | *Streptococcus anginosus* |
|  |  | *Staphylococcus epidermidis* (Moxifloxacin:R) |
| Piperacillin/Tazobactam | 13 | *Escherichia coli* (Piperacillin/Tazobactam:R) (ESBL) |
|  | 3 | *Escherichia coli* (Piperacillin/Tazobactam:R) |
|  | 2 | *Pseudomonas aeruginosa* (Piperacillin/Tazobactam:R) |
|  | 1 | *Enterococcus faecium* (Piperacillin/Tazobactam:R) |
|  |  | *Pseudomonas aeruginosa* (Piperacillin/Tazobactam:R) (Multiresistent strain) |
|  | 1 | *Escherichia coli* (Piperacillin/Tazobactam:R) (ESBL) |
|  |  | *Klebsiella oxytoca* (Piperacillin/Tazobactam:S) |
|  |  | *Proteus mirabilis* (Piperacillin/Tazobactam:S) |
|  |  | *Proteus hauseri* (Piperacillin/Tazobactam:S) |
|  | 1 | *Proteus mirabilis* (Piperacillin/Tazobactam:S) |
|  |  | *Escherichia coli* (Piperacillin/Tazobactam:R) (ESBL) |
|  | 1 | *Pseudomonas aeruginosa* (Piperacillin/Tazobactam:R) |
|  |  | *Staphylococcus epidermidis* |
|  | 1 | *Enterobacter aerogenes* (Piperacillin/Tazobactam: R) |
|  |  | *Klebsiella pneumoniae* (Piperacillin/Tazobactam: R) |
| Piperacillin/Tazobactam, Amikacin | 1 | *Escherichia coli* (Piperacillin/Tazobactam:R, Amikacin:R) (ESBL) |
|  | 1 | *Staphylococcus epidermidis* (Amikacin:R) |
| Piperacillin/Tazobactam, Azithromycin | 1 | *Streptococcus agalactiae* (Piperacillin/Tazobactam:S, Azithromycin:R) |
| Piperacillin/Tazobactam, Erythromycin | 1 | *Staphylococcus epidermidis* (Erythromycin:R) |
|  |  | *Staphylococcus lugdunensis* (Erythromycin:S) |
| Piperacillin/Tazobactam, Gentamicin | 1 | *Acinetobacter baumannii* (Gentamicin:R) (Imipenem and Meropenem resistant strain) |
|  | 1 | *Escherichia coli* (Piperacillin/Tazobactam:R, Gentamicin:S) (ESBL) |
|  |  | *Escherichia coli* (Piperacillin/Tazobactam:R, Gentamicin:S) |
|  |  | *Proteus mirabilis* (Piperacillin/Tazobactam:S, Gentamicin:S) |
| Vancomycin, Amoxicillin/clavulanic acid | 1 | *Klebsiella aerogenes* (Amoxicillin/clavulanic acid:R) |
|  |  | *Klebsiella pneumoniae* (Amoxicillin/clavulanic acid:S) |
| Vancomycin, Clindamycin | 1 | *Escherichia coli* |
|  |  | *Staphylococcus hominis* (Clindamycin:R, Vancomycin:S) |
| Vancomycin, Clindamycin, Amoxicillin/clavulanic acid, Erythromycin | 1 | *Enterococcus faecium* (Amoxicillin/clavulanic acid:R, Vancomycin:S) |
|  |  | *Escherichia coli* (Amoxicillin/clavulanic acid:S) |
|  |  | *Staphylococcus aureus* (Amoxicillin/clavulanic acid:S, Erythromycin:S, Clindamycin:S) |
|  |  | *Staphylococcus simulans* (Amoxicillin/clavulanic acid:S, Erythromycin:S, Clindamycin:S) |
|  |  | *Streptococcus salivarius* (Amoxicillin/clavulanic acid:S, Clindamycin:S) |
| Vancomycin, Moxifloxacin, Penicillin | 1 | *Staphylococcus epidermidis* (Vancomycin:S, Moxifloxacin:R) |
|  | **165** | R = resistant; S = susceptible; |
|  |  | colors: red: inadequate -; yellow: unclear adequacy of -; green: adequate antibiotic therapy |

*Supplementary Methods 1*

The antibiotic therapy noted as free text in the electronic patient files was analyzed by keyword search. In the first step, 537 cases were manually checked, and any spelling errors were included in the keyword search. In addition, all cases with positive blood cultures but no antibiotic therapy at all according to the keyword search were checked manually again, and any spelling variants found were added to the keyword search.
