# Supplementary material for: Empiric antibiotic therapy resistance and mortality in emergency department patients with bloodstream infection: a retrospective cohort study
Source: BMC Emerg Med. 2025 Jan 27;25:18. doi: 10.1186/s12873-025-01177-0 (PMC11773878; doi:10.1186/s12873-025-01177-0)
Supplement: Supplementary file 2 — Supplementary Material 2: Supplementary Table 5 Identified species with resistance to at least one ED antibiotic. Supplementary Table 6. Overview of all utilized ICD codes (n = 1,136) with a comparative analysis between E. Coli-positive and non-E. Coli-positive cases and between S. Aureus-positive and non-S. Aureus-positive cases. Supplementary Table 7 Cox proportional hazards multivariable regression models for S. aureus bacteremia and mortality adjusted for A) Age, Sex, ICD Codes: U07, J18, N39 B) Age, Sex, ICD Codes: A41, U07, J18, N39. Cox proportional hazards multivariable regression models for E. coli bacteremia and mortality adjusted for C) Age, Sex, ICD Codes: U07, J18, N39 D) Age, Sex, ICD Codes: A41, U07, J18, N39 [file 12873_2025_1177_MOESM2_ESM.docx]

|  | ***Acinetobacter baumannii* (n = 1)** | ***Campylobacter jejuni* (n = 1)** | ***Corynebacterium imitans* (n = 3)** | ***Corynebacterium mucifaciens* (n = 3)** | ***Dietzia sp.* (n = 1)** | ***Enterobacter aerogenes***  ***(n = 1)*** | ***Enterobacter cloacae (n = 8)*** | ***Enterococcus faecium* (n = 7)** | ***Escherichia coli* (n = 343)** | ***Klebsiella aerogenes* (n = 4)** | ***Klebsiella oxytoca* (n = 10)** | ***Klebsiella pneumoniae* (n = 75)** | ***Morganella morganii (n = 9)*** | ***Paenibacillus* (n = 1)** | ***Propionibacterium acnes* (n = 10)** | ***Pseudomonas aeruginosa* (n = 40)** | ***Staphylococcus aureus* (n = 142)** | ***Staphylococcus capitis* (n = 18)** | ***Staphylococcus caprae* (n = 4)** | ***Staphylococcus epidermidis* (n = 124)** | ***Staphylococcus haemolyticus* (n = 13)** | ***Staphylococcus hominis* (n = 117)** | ***Staphylococcus pettenkoferi* (n = 7)** | ***Staphylococcus simulans* (n = 5)** | ***Staphylococcus warneri* (n = 6)** | ***Staphylococcus xylosus* (n = 2)** | ***Streptococcus agalactiae* (n = 15)** | ***Streptococcus dysgalactiae* (n = 20)** | ***Streptococcus pneumoniae* (n = 59)** |
| --- | --- | --- | --- | --- | --- | --- | --- | --- | --- | --- | --- | --- | --- | --- | --- | --- | --- | --- | --- | --- | --- | --- | --- | --- | --- | --- | --- | --- | --- |
| **Amikacin** | - | - | - | - | - | - | 0/1 (0%) | - | 1/2 (50%) *ESBL: 1* | - | - | - | - | - | - | - | 0/1 (0%) | - | - | 1/1 (100%) | - | - | - | - | - | - | - | N.A./1 | - |
| **Amoxicillin/ clavulanic acid** | - | - | - | 1/1 (100%) | - | - | - | 1/1 (100%) | 3/5 (60%) *ESBL: 0* | 1/1 (100%) | - | 0/4 (0%) | 1/1 (100%) | - | - | N.A./2 | 2/8 (25%) *MRSA: 2* | - | - | 4/6 (66.7%) | - | 1/3 (33.3%) | - | 0/1 (0%) | - | 0/1 (0%) | - | 0/3 (0%) | - |
| **Ampicillin/ sulbactam** | - | - | - | - | - | - | - | - | 1/2 (50%) *ESBL: 0* | - | - | - | - | - | - | - | N.A./5 | - | - | N.A./3 | - | N.A./2 | - | - | - | - | 0/2 (0%) | 0/1 (0%) | - |
| **Azithromycin** | - | - | 1/1 (100%) | N.A./1 | - | - | - | - | N.A./13 | - | - | N.A./2 | N.A./1 | - | - | N.A./1 | 1/13 (7.7%) *MRSA: 1* | 0/2 (0%) | 1/1 (100%) | 5/9 (55.6%) | - | 5/13 (38.5%) | 1/1 (100%) | - | - | - | 1/1 (100%) | - | 0/4 (0%) |
| **Cefazolin** | - | - | - | - | - | - | - | - | - | - | - | - | - | - | - | - | 0/1 (0%) | - | - | - | - | - | - | - | - | - | - | - | - |
| **Ceftriaxone** | - | - | N.A./2 | N.A./1 | 1/1 (100%) | - | 1/2 (50%) | N.A./1 | 30/153 (19.6%) *ESBL: 29* | 2/2 (100%) | 0/2 (0%) | 6/30 (20%) *ESBL: 6* | 0/3 (0%) | 1/1 (100%) | N.A./3 | N.A./10 | N.A./60 | N.A./8 | N.A./2 | N.A./60 | N.A./7 | N.A./65 | N.A./3 | N.A./3 | N.A./3 | N.A./1 | 0/5 (0%) | 0/8 (0%) | 0/44 (0%) |
| **Cefuroxime** | - | - | - | - | - | - | - | - | - | - | - | - | - | - | - | - | - | - | - | - | - | 1/1 (100%) | - | - | - | - | - | - | - |
| **Ciprofloxacin** | - | 1/1 (100%) | - | - | - | - | - | - | 4/11 (36.4%) *ESBL: 2* | - | - | 2/3 (66.7%) *ESBL: 1* | - | - | N.A./1 | - | 0/1 (0%) | 1/3 (33.3%) | - | 2/2 (100%) | - | 1/3 (33.3%) | - | - | - | - | - | - | - |
| **Clarithromycin** | - | - | N.A./1 | N.A./1 | 0/1 (0%) | - | N.A./1 | N.A./1 | N.A./31 | N.A./1 | - | N.A./6 | - | - | N.A./3 | N.A./2 | 5/21 (23.8%) *MRSA: 0* | 1/3 (33.3%) | 1/1 (100%) | 15/29 (51.7%) | 1/3  (33.3%) | 16/22 (72.7%) | 0/1 (0%) | 1/2 (50%) | 1/2 (50%) | - | 0/1 (0%) | 1/3 (33.3%) | 2/31 (6.5%) |
| **Clindamycin** | - | - | - | - | - | - | - | N.A./1 | N.A./9 | - | - | N.A./1 | - | - | - | N.A./1 | 0/6 (0%) | 0/1 (0%) | - | 2/4 (50%) | - | 2/5 (40%) | - | 0/1 (0%) | - | 1/1 (100%) | 1/3 (33.3%) | 0/2 (0%) | 0/2 (0%) |
| **Cotrimoxazol** | - | - | - | - | - | - | - | - | - | - | - | - | - | - | - | - | - | - | - | - | - | - | - | - | - | - | - | - | - |
| **Erythromycin** | - | - | - | - | - | - | - | N.A./1 | N.A./1 | - | - | - | N.A./1 | - | - | - | 0/1 (0%) | - | - | 1/1 (100%) | - | - | - | 0/1 (0%) | - | - | - | - | - |
| **Gentamicin** | 1/1 (100%) | - | - | - | - | - | - | - | 0/4 (0%) | - | - | 0/1 (0%) | - | - | - | 0/2 (0%) | 0/4 (0%) | 0/1 (0%) | - | 0/2 (0%) | - | 0/1 (0%) | - | - | - | - | - | - | N.A./1 |
| **Imipenem** | - | - | - | - | - | - | 0/1 (0%) | 0/1 (0%) | 0/34 (0%) | - | 0/2 (0%) | 0/10 (0%) | N.A./3 | - | 0/4 (0%) | 0/5 (0%) | N.A./10 | N.A./1 | - | N.A./9 | - | N.A./9 | - | - | - | - | 0/1 (0%) | 0/1 (0%) | - |
| **Levofloxacin** | - | - | - | - | - | - | - | - | - | - | - | 1/1 (100%) *ESBL: 1* | - | - | - | - | - | - | - | - | - | 1/1 (100%) | - | - | - | - | - | - | - |
| **Meropenem** | - | - | - | - | - | - | 0/3 (0%) | N.A./3 | 0/61 (0%) | 0/1 (0%) | 0/3 (0%) | 0/18 (0%) | - | - | - | 1/10 (10%) | N.A./27 | N.A./3 | - | N.A./11 | N.A./3 | N.A./11 | N.A./3 | - | - | - | 0/1 (0%) | 0/2 (0%) | 0/5 (0%) |
| **Metronidazole** | - | N.A./1 | - | - | - | - | - | N.A./1 | N.A./17 | N.A./1 | N.A./2 | N.A./2 | - | - | 2/2 (100%) | N.A./1 | - | N.A./2 | - | N.A./4 | N.A./1 | N.A./3 | N.A./1 | - | - | - | - | - | - |
| **Moxifloxacin** | - | - | - | - | - | - | - | - | 0/3 (0%) | - | 1/1 (100%) | 0/1 (0%) | - | - | N.A./1 | N.A./1 | 0/1 (0%) | - | - | 3/4 (75%) | - | - | - | - | - | - | 0/1 (0%) | - | 0/3 (0%) |
| **Penicillin** | - | - | - | - | - | - | - | - | - | - | - | - | - | - | - | - | N.A./2 | - | - | 0/1  (0 %) | - | - | - | - | - | - | - | 0/1 (0%) | - |
| **Piperacillin/ Tazobactam** | N.A./1 | - | N.A./1 | N.A./1 | - | 1/1 (100%) | 0/3 (0%) | 1/1 (100%) | 20/78 (25.6%) *ESBL: 17* | - | 0/2 (0%) | 1/10 (10%) *ESBL: 1* | 0/1 (0%) | - | 0/1 (0%) | 4/12 (33.3%) | N.A./27 | N.A./3 | N.A./2 | N.A./28 | N.A./3 | N.A./21 | N.A./1 | N.A./1 | N.A./3 | N.A./1 | 0/4 (0%) | 0/3 (0%) | 0/7 (0%) |
| **Vancomycin** | - | - | - | - | - | - | - | 0/1 (0%) | N.A./13 | N.A./1 | - | N.A./7 | - | - | - | N.A./4 | 0/23 (0%) | N.A./1 | - | N.A./11 | N.A./1 | N.A./7 | N.A./1 | 0/1 (0%) | - | - | N.A./4 | N.A./4 | N.A./3 |
| **Overall resistance against at least one given antibiotic** | **1 (100%)** | **1 (100%)** | **1 (33.3%)** | **1 (33.3%)** | **1 (100%)** | **1 (100%)** | **1 (12.5%)** | **2 (28.6%)** | **58 (16.9%)** | **3  (75%)** | **1  (10%)** | **10**  **(13.3%)** | **1 (11.1%)** | **1 (100%)** | **2  (20%)** | **5 (12.5%)** | **8  (5.6%)** | **2 (11.1%)** | **2  (50%)** | **33 (26.6%)** | **1  (7.7%)** | **27 (23.1%)** | **1 (14.3%)** | **1  (20%)** | **1 (16.7%)** | **1  (50%)** | **2 (13.3%)** | **1  (5%)** | **2  (3.4%)** |
| **Resistant strain ESBL or MRSA** | **-** | **-** | **-** | **-** | **-** |  | **-** | **-** | **49 (84.5%)** | **-** | **-** | **9**  **(90%)** | **-** | **-** | **-** | **-** | **3 (37.5%)** | **-** | **-** | **-** | **-** | **-** | **-** | **-** | **-** | **-** | **-** | **-** | **-** |
| **Notation used: r/a (%) r = number of cases with bacterial resistance against the given antibiotic; a = total number of cases with bacterial presence and receiving the defined antibiotic; N.A. = antibiotic was not part of the antibiogram of this bacterium**  **The number of ESBL and MRSA cases resistant to AB are shown in *italic*. Cells, which are showing resistance, are highlighted.** | | | | | | | | | | | | | | | | | | | | | | | | | | | | | |

**Supplementary Table 5** Identified species with resistance to at least one ED antibiotic.

**Supplementary Table 6.** Overview of all registered ICD codes (n = 1,136) with a comparative analysis between *E. coli*-positive and *E. coli*-negative cases and between *S. aureus*-positive and *S. aureus*-negative cases.

| ICD Code | all cases  (n= 1136) | | Comparison by *E. coli* positivity | | | | Comparison by *S. aureus* positivity | | | |
| --- | --- | --- | --- | --- | --- | --- | --- | --- | --- | --- |
|  |  |  | *E. coli*-positive  (n= 343) | | *E. coli*-negative  (n= 793) | | *S. aureus*-positive  (n= 142) | | *S.aureus*-negative (n= 994) | |
|  | n | % | n | % | n | % | n | % | n | % |
| A41 | 568 | 50.0% | 175 | 51.0% | 393 | 49.6% | 92 | 64.8% | 476 | 47.9% |
| N39 | 223 | 19.6% | 106 | 30.9% | 117 | 14.8% | 14 | 9.9% | 209 | 21.0% |
| J18 | 221 | 19.5% | 40 | 11.7% | 181 | 22.8% | 25 | 17.6% | 196 | 19.7% |
| E86 | 211 | 18.6% | 67 | 19.5% | 144 | 18.2% | 29 | 20.4% | 182 | 18.3% |
| J96 | 152 | 13.4% | 30 | 8.7% | 122 | 15.4% | 16 | 11.3% | 136 | 13.7% |
| R50 | 113 | 9.9% | 40 | 11.7% | 73 | 9.2% | 11 | 7.7% | 102 | 10.3% |
| U07 | 107 | 9.4% | 24 | 7.0% | 83 | 10.5% | 20 | 14.1% | 87 | 8.8% |
| R10 | 100 | 8.8% | 46 | 13.4% | 54 | 6.8% | 6 | 4.2% | 94 | 9.5% |
| R06 | 80 | 7.0% | 10 | 2.9% | 70 | 8.8% | 13 | 9.2% | 67 | 6.7% |
| I50 | 58 | 5.1% | 14 | 4.1% | 44 | 5.5% | 4 | 2.8% | 54 | 5.4% |
| D64 | 40 | 3.5% | 10 | 2.9% | 30 | 3.8% | 6 | 4.2% | 34 | 3.4% |
| I10 | 38 | 3.3% | 12 | 3.5% | 26 | 3.3% | 2 | 1.4% | 36 | 3.6% |
| J44 | 32 | 2.8% | 8 | 2.3% | 24 | 3.0% | 0 | 0.0% | 32 | 3.2% |
| I48 | 30 | 2.6% | 9 | 2.6% | 21 | 2.6% | 2 | 1.4% | 28 | 2.8% |
| N18 | 27 | 2.4% | 6 | 1.7% | 21 | 2.6% | 10 | 7.0% | 17 | 1.7% |
| N30 | 27 | 2.4% | 11 | 3.2% | 16 | 2.0% | 3 | 2.1% | 24 | 2.4% |
| J15 | 26 | 2.3% | 8 | 2.3% | 18 | 2.3% | 1 | 0.7% | 25 | 2.5% |
| R41 | 25 | 2.2% | 7 | 2.0% | 18 | 2.3% | 3 | 2.1% | 22 | 2.2% |
| E11 | 24 | 2.1% | 11 | 3.2% | 13 | 1.6% | 3 | 2.1% | 21 | 2.1% |
| E14 | 19 | 1.7% | 6 | 1.7% | 13 | 1.6% | 4 | 2.8% | 15 | 1.5% |
| K83 | 19 | 1.7% | 11 | 3.2% | 8 | 1.0% | 0 | 0.0% | 19 | 1.9% |
| K92 | 16 | 1.4% | 5 | 1.5% | 11 | 1.4% | 4 | 2.8% | 12 | 1.2% |
| Z59 | 15 | 1.3% | 0 | 0.0% | 15 | 1.9% | 1 | 0.7% | 14 | 1.4% |
| A46 | 14 | 1.2% | 0 | 0.0% | 14 | 1.8% | 2 | 1.4% | 12 | 1.2% |
| E10 | 14 | 1.2% | 0 | 0.0% | 14 | 1.8% | 2 | 1.4% | 12 | 1.2% |
| N17 | 13 | 1.1% | 7 | 2.0% | 6 | 0.8% | 1 | 0.7% | 12 | 1.2% |
| R11 | 13 | 1.1% | 5 | 1.5% | 8 | 1.0% | 2 | 1.4% | 11 | 1.1% |
| I25 | 12 | 1.1% | 2 | 0.6% | 10 | 1.3% | 3 | 2.1% | 9 | 0.9% |
| K80 | 12 | 1.1% | 8 | 2.3% | 4 | 0.5% | 0 | 0.0% | 12 | 1.2% |
| L89 | 11 | 1.0% | 2 | 0.6% | 9 | 1.1% | 1 | 0.7% | 10 | 1.0% |
| L97 | 11 | 1.0% | 2 | 0.6% | 9 | 1.1% | 3 | 2.1% | 8 | 0.8% |
| G40 | 10 | 0.9% | 2 | 0.6% | 8 | 1.0% | 1 | 0.7% | 9 | 0.9% |
| I95 | 10 | 0.9% | 4 | 1.2% | 6 | 0.8% | 0 | 0.0% | 10 | 1.0% |
| J12 | 10 | 0.9% | 3 | 0.9% | 7 | 0.9% | 1 | 0.7% | 9 | 0.9% |
| R52 | 10 | 0.9% | 1 | 0.3% | 9 | 1.1% | 4 | 2.8% | 6 | 0.6% |
| B34 | 9 | 0.8% | 2 | 0.6% | 7 | 0.9% | 2 | 1.4% | 7 | 0.7% |
| E87 | 9 | 0.8% | 3 | 0.9% | 6 | 0.8% | 1 | 0.7% | 8 | 0.8% |
| I26 | 9 | 0.8% | 0 | 0.0% | 9 | 1.1% | 0 | 0.0% | 9 | 0.9% |
| K81 | 8 | 0.7% | 2 | 0.6% | 6 | 0.8% | 1 | 0.7% | 7 | 0.7% |
| R07 | 8 | 0.7% | 2 | 0.6% | 6 | 0.8% | 1 | 0.7% | 7 | 0.7% |
| R73 | 8 | 0.7% | 3 | 0.9% | 5 | 0.6% | 1 | 0.7% | 7 | 0.7% |
| G03 | 7 | 0.6% | 0 | 0.0% | 7 | 0.9% | 1 | 0.7% | 6 | 0.6% |
| L08 | 7 | 0.6% | 1 | 0.3% | 6 | 0.8% | 2 | 1.4% | 5 | 0.5% |
| N23 | 7 | 0.6% | 4 | 1.2% | 3 | 0.4% | 0 | 0.0% | 7 | 0.7% |
| R33 | 7 | 0.6% | 1 | 0.3% | 6 | 0.8% | 0 | 0.0% | 7 | 0.7% |
| D38 | 6 | 0.5% | 2 | 0.6% | 4 | 0.5% | 0 | 0.0% | 6 | 0.6% |
| D46 | 6 | 0.5% | 2 | 0.6% | 4 | 0.5% | 1 | 0.7% | 5 | 0.5% |
| I21 | 6 | 0.5% | 1 | 0.3% | 5 | 0.6% | 2 | 1.4% | 4 | 0.4% |
| I63 | 6 | 0.5% | 1 | 0.3% | 5 | 0.6% | 1 | 0.7% | 5 | 0.5% |
| I64 | 6 | 0.5% | 0 | 0.0% | 6 | 0.8% | 2 | 1.4% | 4 | 0.4% |
| K57 | 6 | 0.5% | 1 | 0.3% | 5 | 0.6% | 1 | 0.7% | 5 | 0.5% |
| K71 | 6 | 0.5% | 3 | 0.9% | 3 | 0.4% | 0 | 0.0% | 6 | 0.6% |
| K85 | 6 | 0.5% | 2 | 0.6% | 4 | 0.5% | 0 | 0.0% | 6 | 0.6% |
| N10 | 6 | 0.5% | 1 | 0.3% | 5 | 0.6% | 2 | 1.4% | 4 | 0.4% |
| N11 | 6 | 0.5% | 4 | 1.2% | 2 | 0.3% | 0 | 0.0% | 6 | 0.6% |
| N19 | 6 | 0.5% | 1 | 0.3% | 5 | 0.6% | 1 | 0.7% | 5 | 0.5% |
| R05 | 6 | 0.5% | 0 | 0.0% | 6 | 0.8% | 0 | 0.0% | 6 | 0.6% |
| R57 | 6 | 0.5% | 1 | 0.3% | 5 | 0.6% | 0 | 0.0% | 6 | 0.6% |
| C18 | 5 | 0.4% | 2 | 0.6% | 3 | 0.4% | 1 | 0.7% | 4 | 0.4% |
| F03 | 5 | 0.4% | 2 | 0.6% | 3 | 0.4% | 0 | 0.0% | 5 | 0.5% |
| I69 | 5 | 0.4% | 0 | 0.0% | 5 | 0.6% | 1 | 0.7% | 4 | 0.4% |
| K70 | 5 | 0.4% | 2 | 0.6% | 3 | 0.4% | 1 | 0.7% | 4 | 0.4% |
| M86 | 5 | 0.4% | 0 | 0.0% | 5 | 0.6% | 1 | 0.7% | 4 | 0.4% |
| T68 | 5 | 0.4% | 1 | 0.3% | 4 | 0.5% | 0 | 0.0% | 5 | 0.5% |
| A04 | 4 | 0.4% | 2 | 0.6% | 2 | 0.3% | 0 | 0.0% | 4 | 0.4% |
| A09 | 4 | 0.4% | 0 | 0.0% | 4 | 0.5% | 0 | 0.0% | 4 | 0.4% |
| C92 | 4 | 0.4% | 3 | 0.9% | 1 | 0.1% | 0 | 0.0% | 4 | 0.4% |
| I42 | 4 | 0.4% | 0 | 0.0% | 4 | 0.5% | 2 | 1.4% | 2 | 0.2% |
| K72 | 4 | 0.4% | 3 | 0.9% | 1 | 0.1% | 0 | 0.0% | 4 | 0.4% |
| K75 | 4 | 0.4% | 0 | 0.0% | 4 | 0.5% | 0 | 0.0% | 4 | 0.4% |
| N13 | 4 | 0.4% | 4 | 1.2% | 0 | 0.0% | 0 | 0.0% | 4 | 0.4% |
| N20 | 4 | 0.4% | 4 | 1.2% | 0 | 0.0% | 0 | 0.0% | 4 | 0.4% |
| R40 | 4 | 0.4% | 1 | 0.3% | 3 | 0.4% | 0 | 0.0% | 4 | 0.4% |
| R55 | 4 | 0.4% | 1 | 0.3% | 3 | 0.4% | 2 | 1.4% | 2 | 0.2% |
| Z95 | 4 | 0.4% | 1 | 0.3% | 3 | 0.4% | 1 | 0.7% | 3 | 0.3% |
| A49 | 3 | 0.3% | 1 | 0.3% | 2 | 0.3% | 0 | 0.0% | 3 | 0.3% |
| D69 | 3 | 0.3% | 2 | 0.6% | 1 | 0.1% | 0 | 0.0% | 3 | 0.3% |
| E16 | 3 | 0.3% | 2 | 0.6% | 1 | 0.1% | 1 | 0.7% | 2 | 0.2% |
| J20 | 3 | 0.3% | 2 | 0.6% | 1 | 0.1% | 0 | 0.0% | 3 | 0.3% |
| J90 | 3 | 0.3% | 1 | 0.3% | 2 | 0.3% | 1 | 0.7% | 2 | 0.2% |
| J98 | 3 | 0.3% | 0 | 0.0% | 3 | 0.4% | 2 | 1.4% | 1 | 0.1% |
| K50 | 3 | 0.3% | 0 | 0.0% | 3 | 0.4% | 1 | 0.7% | 2 | 0.2% |
| K52 | 3 | 0.3% | 2 | 0.6% | 1 | 0.1% | 0 | 0.0% | 3 | 0.3% |
| K56 | 3 | 0.3% | 1 | 0.3% | 2 | 0.3% | 0 | 0.0% | 3 | 0.3% |
| K76 | 3 | 0.3% | 1 | 0.3% | 2 | 0.3% | 0 | 0.0% | 3 | 0.3% |
| M00 | 3 | 0.3% | 0 | 0.0% | 3 | 0.4% | 1 | 0.7% | 2 | 0.2% |
| M54 | 3 | 0.3% | 1 | 0.3% | 2 | 0.3% | 0 | 0.0% | 3 | 0.3% |
| R18 | 3 | 0.3% | 1 | 0.3% | 2 | 0.3% | 0 | 0.0% | 3 | 0.3% |
| R31 | 3 | 0.3% | 2 | 0.6% | 1 | 0.1% | 0 | 0.0% | 3 | 0.3% |
| Z92 | 3 | 0.3% | 1 | 0.3% | 2 | 0.3% | 1 | 0.7% | 2 | 0.2% |
| C34 | 2 | 0.2% | 0 | 0.0% | 2 | 0.3% | 0 | 0.0% | 2 | 0.2% |
| C91 | 2 | 0.2% | 1 | 0.3% | 1 | 0.1% | 0 | 0.0% | 2 | 0.2% |
| F05 | 2 | 0.2% | 1 | 0.3% | 1 | 0.1% | 1 | 0.7% | 1 | 0.1% |
| G35 | 2 | 0.2% | 0 | 0.0% | 2 | 0.3% | 0 | 0.0% | 2 | 0.2% |
| G82 | 2 | 0.2% | 0 | 0.0% | 2 | 0.3% | 1 | 0.7% | 1 | 0.1% |
| I46 | 2 | 0.2% | 1 | 0.3% | 1 | 0.1% | 0 | 0.0% | 2 | 0.2% |
| I49 | 2 | 0.2% | 0 | 0.0% | 2 | 0.3% | 0 | 0.0% | 2 | 0.2% |
| I61 | 2 | 0.2% | 1 | 0.3% | 1 | 0.1% | 1 | 0.7% | 1 | 0.1% |
| I70 | 2 | 0.2% | 2 | 0.6% | 0 | 0.0% | 0 | 0.0% | 2 | 0.2% |
| I74 | 2 | 0.2% | 1 | 0.3% | 1 | 0.1% | 1 | 0.7% | 1 | 0.1% |
| K59 | 2 | 0.2% | 1 | 0.3% | 1 | 0.1% | 0 | 0.0% | 2 | 0.2% |
| K65 | 2 | 0.2% | 2 | 0.6% | 0 | 0.0% | 0 | 0.0% | 2 | 0.2% |
| L03 | 2 | 0.2% | 2 | 0.6% | 0 | 0.0% | 1 | 0.7% | 1 | 0.1% |
| M79 | 2 | 0.2% | 0 | 0.0% | 2 | 0.3% | 2 | 1.4% | 0 | 0.0% |
| R25 | 2 | 0.2% | 1 | 0.3% | 1 | 0.1% | 1 | 0.7% | 1 | 0.1% |
| R39 | 2 | 0.2% | 2 | 0.6% | 0 | 0.0% | 0 | 0.0% | 2 | 0.2% |
| R42 | 2 | 0.2% | 0 | 0.0% | 2 | 0.3% | 0 | 0.0% | 2 | 0.2% |
| R51 | 2 | 0.2% | 0 | 0.0% | 2 | 0.3% | 0 | 0.0% | 2 | 0.2% |
| R60 | 2 | 0.2% | 1 | 0.3% | 1 | 0.1% | 1 | 0.7% | 1 | 0.1% |
| A08 | 1 | 0.1% | 0 | 0.0% | 1 | 0.1% | 0 | 0.0% | 1 | 0.1% |
| F10 | 1 | 0.1% | 0 | 0.0% | 1 | 0.1% | 0 | 0.0% | 1 | 0.1% |
| G20 | 1 | 0.1% | 0 | 0.0% | 1 | 0.1% | 0 | 0.0% | 1 | 0.1% |
| G41 | 1 | 0.1% | 1 | 0.3% | 0 | 0.0% | 0 | 0.0% | 1 | 0.1% |
| G45 | 1 | 0.1% | 1 | 0.3% | 0 | 0.0% | 0 | 0.0% | 1 | 0.1% |
| G93 | 1 | 0.1% | 1 | 0.3% | 0 | 0.0% | 0 | 0.0% | 1 | 0.1% |
| J06 | 1 | 0.1% | 0 | 0.0% | 1 | 0.1% | 0 | 0.0% | 1 | 0.1% |
| J85 | 1 | 0.1% | 1 | 0.3% | 0 | 0.0% | 0 | 0.0% | 1 | 0.1% |
| K29 | 1 | 0.1% | 0 | 0.0% | 1 | 0.1% | 0 | 0.0% | 1 | 0.1% |
| N45 | 1 | 0.1% | 0 | 0.0% | 1 | 0.1% | 0 | 0.0% | 1 | 0.1% |
| R02 | 1 | 0.1% | 0 | 0.0% | 1 | 0.1% | 1 | 0.7% | 0 | 0.0% |
| R04 | 1 | 0.1% | 0 | 0.0% | 1 | 0.1% | 0 | 0.0% | 1 | 0.1% |
| R17 | 1 | 0.1% | 0 | 0.0% | 1 | 0.1% | 0 | 0.0% | 1 | 0.1% |
| R45 | 1 | 0.1% | 1 | 0.3% | 0 | 0.0% | 0 | 0.0% | 1 | 0.1% |
| R47 | 1 | 0.1% | 1 | 0.3% | 0 | 0.0% | 0 | 0.0% | 1 | 0.1% |
| R72 | 1 | 0.1% | 0 | 0.0% | 1 | 0.1% | 0 | 0.0% | 1 | 0.1% |
| R82 | 1 | 0.1% | 0 | 0.0% | 1 | 0.1% | 0 | 0.0% | 1 | 0.1% |

**Supplementary Table 7.** Cox proportional hazards multivariable regression models for *S. aureus* bacteremia and mortality adjusted for A) Age, Sex, ICD Codes: U07, J18, N39 B) Age, Sex, ICD Codes: A41, U07, J18, N39. Cox proportional hazards multivariable regression models for E*. coli* bacteremia and mortality adjusted for C) Age, Sex, ICD Codes: U07, J18, N39 D) Age, Sex, ICD Codes: A41, U07, J18, N39.

| Variables | ***S. aureus -* positivity** | | | | ***E. coli -* positivity** | | | |
| --- | --- | --- | --- | --- | --- | --- | --- | --- |
|  | Model A) | | Model B) | | Model C) | | Model D) | |
|  | HR (95% CI) | p-value | HR (95% CI) | p-value | HR (95% CI) | p-value | HR (95% CI) | p-value |
| Age | 1.031 (1.025 - 1.038) | **< 0.001** | 1.030 (1.024 - 1.037) | **< 0.001** | 1.032 (1.025 - 1.038) | **< 0.001** | 1.031 (1.024 - 1.037) | **< 0.001** |
| Male Gender | 1.107 (0.948 - 1.294) | 0.199 | 1.095 (0.937 - 1.279) | 0.254 | 1.065 (0.911 - 1.246) | 0.430 | 1.050 (0.897 - 1.228) | 0.546 |
| A41 Sepsis | - | - | 1.234 (1.053 - 1.447) | **0.010** | - | - | 1.265 (1.081 - 1.480) | **0.003** |
| U07 Covid-19 | 1.029 (0.797 - 1.328) | 0.827 | 1.056 (0.818 - 1.365) | 0.674 | 1.040 (0.806 - 1.343) | 0.760 | 1.068 (0.827 - 1.380) | 0.612 |
| J18 Pneumonia | 0.995 (0.823 - 1.202) | 0.957 | 1.057 (0.870 - 1.284) | 0.576 | 0.933 (0.771 - 1.128) | 0.472 | 0.996 (0.820 - 1.211) | 0.969 |
| N39 UTI | 0.665 (0.543 - 0.814) | **< 0.001** | 0.672 (0.549 - 0.823) | **< 0.001** | 0.697 (0.569 - 0.855) | **< 0.001** | 0.706 (0.576 - 0.865) | **< 0.001** |
| *S. aureus* positivity | 1.375 (1.098 - 1.721) | **0.006** | 1.319 (1.052 - 1.655) | **0.017** | - | - | - | - |
| *E. coli* positivity | - | - | - | - | 0.668 (0.557 - 0.800) | **< 0.001** | 0.667 (0.557 - 0.799) | **< 0.001** |
